# Ginsenoside Rb1 Attenuates Oxygen-Glucose Deprivation-Induced Apoptosis in SH-SY5Y Cells via Protection of Mitochondria and Inhibition of AIF and Cytochrome c Release

**DOI:** 10.3390/molecules181012777

**Published:** 2013-10-16

**Authors:** Jianmin Liang, Ying Yu, Boyu Wang, Bin Lu, Jizhou Zhang, Hongbo Zhang, Pengfei Ge

**Affiliations:** 1 Department of Pediatrics, First Hospital of Jilin University, Changchun 130021, China; E-Mails: jackyliangjm@163.com (J.L.); zhb815656@sina.com (H.Z.); 2 Department of Neurosurgery, First Hospital of Jilin University, Changchun 130021, China; E-Mails: yuying3210@sina.com (Y.Y.); drwangboyu@163.com (B.W.); lubin03162@126.com (B.L.); 3 Department of Biochemistry, Bethune Medical School of Jilin University, Changchun 130021, China; E-Mail: zhangjizhou7601@sina.com

**Keywords:** ginsenoside Rb1, oxygen-glucose deprivation, mitochondria, apoptosis inducing factor, cytochrome c

## Abstract

To investigate the role of mitochondria in the protective effects of ginsenoside Rb1 on cellular apoptosis caused by oxygen-glucose deprivation, in this study, MTT assay, TUNEL staining, flow cytometry, immunocytochemistry and western blotting were used to examine the cellular viability, apoptosis, ROS level, mitochondrial membrane potential, and the distribution of apoptosis inducing factor, cytochrome c, Bax and Bcl-2 in nucleus, mitochondria and cytoplasm. We found that pretreatment with GRb1 improved the cellular viability damaged by OGD. Moreover, GRb1 inhibited apoptosis in SH-SY5Y cells induced by OGD. Further studies showed that the elevation of cellular reactive oxygen species levels and the reduction of mitochondrial membrane potential caused by OGD were both counteracted by GRb1. Additionally, GRb1 not only suppressed the translocation of apoptosis inducing factor into nucleus and cytochrome c into cytoplasm, but also inhibited the increase of Bax within mitochondria and alleviated the decrease of mitochondrial Bcl-2. Our study indicates that the protection of GRb1 on OGD-induced apoptosis in SH-SY5Y cells is associated with its protection on mitochondrial function and inhibition of release of AIF and cytochrome c.

## 1. Introduction

Ischemic stroke due to lack of cerebral blood supply is one of the most common causes leading to death or disability in adults worldwide [1]. However, both animal studies and clinical finding revealed that reperfusion following ischemia results in delayed neuronal injury or death [2,3]. Although the modes of neuronal death caused by cerebral ischemia and reperfusion still need to be elucidated, both *in vivo* and *in vitro* studies have shown that apoptosis is a common form of neuronal death following ischemia and reperfusion [4,5]. Other pathological conditions such as traumatic brain injury or neurodegenerative diseases would cause neuronal apoptosis as well [6,7]. By contrast, suppression of apoptosis has been demonstrated to be the main mechanism underlying the protective effects of some chemicals against neuronal death [8,9]. In addition, it is found that the protective effects of ischemic postconditioning and preconditioning on neuronal death induced by ischemia and reperfusion are associated with inhibition of apoptosis [10]. Thus, these previous studies not only show that apoptosis plays an important role in neuronal death caused by various pathological stresses, but also indicate that anti-apoptosis might be a strategy to prevent or alleviate neuronal damage induced by ischemia and reperfusion. 

Ginseng, the root of *Panax ginseng* C.A. Meyer, has been a very important component of Chinese prescriptions for thousands of years [11]. Until now, part of the components of ginseng have been isolated and over 40 ginsenosides have been identified [11], among which ginsenoside Rb1 (GRb1) has been extensively studied and found to have multiple biological functions including anti-inflammation, anti-apoptosis and induction of neurogenesis properties [12,13,14]. In particular, GRb1 has been demonstrated to inhibit ischemia- and reperfusion-induced cellular death in the heart, liver and brain [15,16,17]. Thus, GRb1 might be a potential medicine used for the treatment of cerebral injury caused by ischemia and reperfusion. 

Recently, it was reported that the protection of GRb1 against neuronal death is correlated with its anti-apoptosis effects [11], despite the fact its underlying mechanism is still elusive. Neuronal apoptosis following cerebral ischemia and reperfusion is found to be related to many factors, such as oxidative stress, endoplasmic reticulum stress, neuroinflammation, and activation of apoptosis associated signal pathways [18,19,20,21]. However, accumulating evidence has shown that the mitochondrion is an important organelle in modulating cellular apoptosis. Exogenous or endogenous stress could make mitochondria lose mitochondrial complex-I activity, depolarize mitochondrial membrane potentials, and release of apoptosis inducing factor (AIF) [22]. Protection of mitochondria has showed anti-apoptotic effects. Despite animal studies showing that GRb1 mitigated neuronal apoptosis caused by ischemia and reperfusion [11], its effects on mitochondrial function are still unclear.

SH-SY5Y cells are human neuroblastoma cells, which are similar to neurons in morphological, neurochemical and electrophysiological properties and have been extensively used as an *in vitro* model to study neuronal injury or death [23]. Oxygen-glucose deprivation (OGD) of SH-SY5Y cells is a well-established and widely used *in vitro* model for ischemic studies [24]. Thus, the present study aimed to determine whether ginsenoside Rb1 protects against SH-SY5Y apoptosis caused by OGD via maintaining mitochondrial function. 

## 2. Results

### 2.1. Ginsenoside Rb1 Decreased Cell Death Caused by OGD

**Figure 1:**
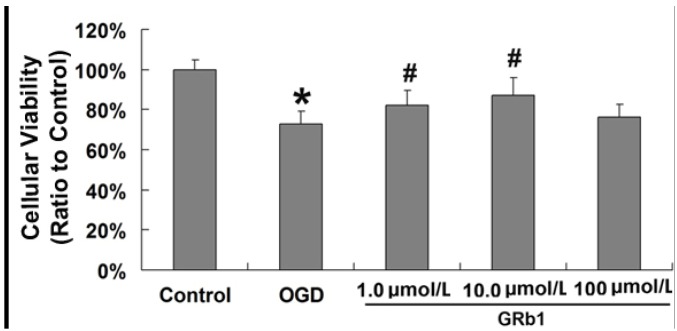
MTT assay of cellular viability. Pretreatment of GRb1 at the concentration of 1.0 µmol/L and 10.0 µmol/L significantly suppressed cellular death caused by OGD at 24h. However, 100 µmol/L GRb1 did not show protection on cellular viability. * *p* < 0.01 *versus* control group; **^#^** *p* < 0.01 *versus* OGD group.

In order to investigate the protective effects of GRb1 on cell death caused by OGD, MTT assay was used to assess cellular viability in SH-SY5Y cells. As shown in Figure 1, cellular viability decreased in SH-SY5Y cells to 68.5% ± 5.2% at 24 h after OGD, but it was reverted to 82.1% ± 5.8% (*p* < 0.01 *versus* OGD group) and 87.3% ± 6.3% (*p* < 0.01 *versus* OGD group), respectively, by treatment with GRb1 at concentrations of 1.0 µmol/L and 10 µmol/L. However, 100 µmol/L GRb1 did not show any protection on cellular viability when compared with that in the OGD group; we thus think this might because a higher concentration of GRb1 might produce toxic effects on SH-SY5Y cells, which counteract its protective effect. This result indicated that treatment with 1.0 µmol/L and 10.0 µmol/L GRb1 protects the viability of SH-SY5Y cells, but 100 µmol/L GRb1 did not. Thus, the concentration of 1.0 µmol/L and 10.0 µmol/L was used in the subsequent experiments to investigate the protective effects of GRb1 on OGD induced cell death. 

### 2.2. Ginsenoside Rb1 Inhibited Apoptosis Caused by OGD

**Figure 2:**
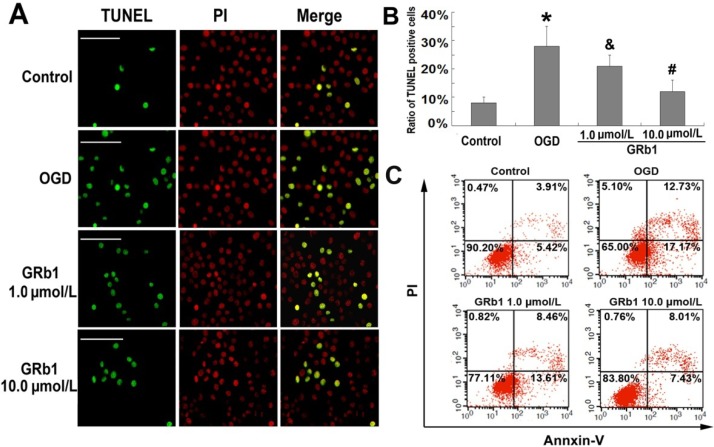
Apoptosis detected by TUNEL staining and flow cytometry. (**A**) representative image of TUNEL staining; (**B**) statistical results; (**C**) flow cytometry. The cells with positive reaction to TUNEL staining showed green color and represented apoptotic cells. * *p* < 0.01 *versus* control group; **^&^** *p* < 0.05 *versus* OGD group; **^#^** *p* < 0.01 *versus* OGD group. Scale bar: 40 µm.

It was reported previously that OGD induced apoptosis in SH-SY5Y cells [25], so we evaluated the protective effects of GRb1 on OGD-induced apoptosis in SH-SY5Y cells. TUNEL staining is often used to assess apoptosis by detecting DNA fragmentation, a typical feature of apoptosis. As Figure 2A,B show, the percentage of positive cells to TUNEL staining (green color) was 28.8 ± 7.1% in the OGD group, which was significantly higher than 7.8% ± 1.9% in the control group. However, the elevated percentage reduced to 20.9% ± 3.8% (*p* < 0.05 *versus* OGD group) and 12.4% ± 4.1% (*p* < 0.01 *versus* OGD group) when the cells were pretreated with GRb1 at the concentrations of 1.0 µmol/L and 10.0 µmol/L, respectively. Further, we used flow cytometry with Annexin V-FITC and PI staining to measure the rate of apoptosis. As shown in Figure 2C, the percentage of SH-SY5Y cells at early stage of apoptosis (Annxin V+/PI−) increased from 5.42% to 17.17%, and the percentage at late stage (Annxin V+/PI+) increased from 3.91% to 12.73% in the OGD group when compared with those in control group. By contrast, pretreatment with 1.0 µmol/L or 10.0 µmol/L GRb1 suppressed the early stage apoptotic rates to 13.61% and 7.43%, respectively. Meanwhile, the percentage of apoptotic cells at late stage was inhibited to 8.46% and 8.01%. Thus, the results form TUNEL staining and flow cytometry indicated that GRb1 inhibited OGD-induced apoptosis in SH-SY5Y cells. 

### 2.3. Ginsenoside Rb1 Attenuated ROS Level

**Figure 3:**
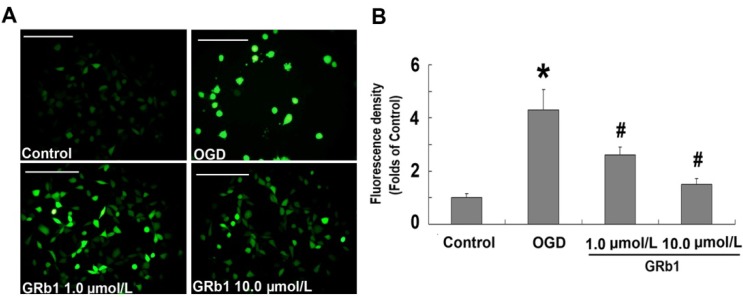
Measurement of ROS level. (**A**) representative images under fluorescence microscope; (**B**) fluorescence density statistics. Fluorescence microscopy showed the fluorescence in the OGD group was significantly higher than that in the control group, but it was suppressed by treatment with either 1.0 µmol/L or 10.0 µmol/L GRb1. Further study showed that the fluorescence density in the OGD group was 4.32 ± 0.76 times as high as that in the control group. By contrast, it was reduced markedly to 2.64 ± 0.31 and 1.5 ± 0.22 times when the cells were treated with 1.0 µmol/L or 10.0 µmol/L GRb1, respectively. This result indicated that GRb1 mitigated the increase of intracellular ROS level caused by OGD. * *p* < 0.01 *versus* control group; **^#^** *p* < 0.01 *versus* OGD group. Scale bar: 50 µm.

The mitochondrion is thought to be the main site where reactive oxygen species (ROS) are produced, and increased ROS levels within the cell reflect mitochondrial dysfunction [26]. Therefore, we examined the effect of GRb1 on ROS levels. As shown in Figure 3A, fluorescence microscopy revealed that the fluorescence density increased significantly in OGD-treated SH-SY5Y cells when compared with the control group. However, the elevated fluorescence was attenuated effectively by GRb1 either at the concentration of 1.0 µmol/L or 10.0 µmol/L. Consistent with the fluorescence microscopy findings, the results from measurement of the fluorescence density showed that the ROS level in the OGD group was 4.32 ± 0.76 times as high as that in the control group (Figure 3B). By contrast, it reduced markedly to 2.64 ± 0.31 and 1.5 ± 0.22 times when the cells were treated with 1.0 µmol/L or 10.0 µmol/L GRb1, respectively. These results indicated that GRb1 could effectively suppress ROS levels induced by OGD in SH-SY5Y cells. Moreover, the higher concentration of GRb1 showed better inhibitory effects on ROS than the lower concentration did.

### 2.4. Ginsenoside Rb1 Sustained Mitochondrial Membrane Potential (ΔΨm)

**Figure 4:**
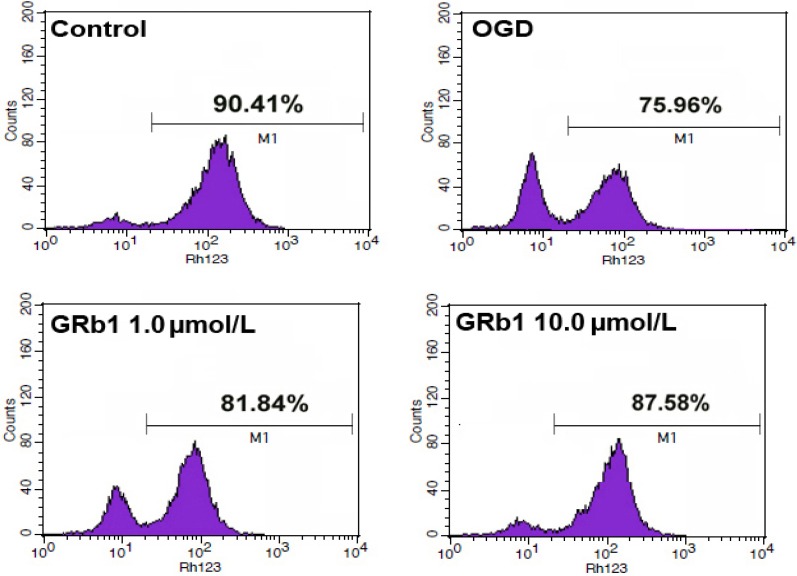
Measurement of mitochondrial membrane potential by flow cytometry. Treatment with 1.0 µmol/L or 10.0 µmol/L GRb1 counteracted OGD-induced reduction of mitochondrial membrane potential in SH-SY5Y cells. This result indicated that GRb1 protected mitochondrial damage caused by OGD.

Mitochondrial dysfunction is characterized by depolarization of mitochondrial membranes [27]. Rhodamine 123, is a cell-permeable cationic dye that preferentially enters the mitochondria based on the highly negative mitochondrial membrane potential. Depolarization of the membrane results in the loss of Rhodamine 123 from the mitochondria and a decrease in intracellular fluorescence. As shown in Figure 4, OGD made the mitochondrial membrane potentials of SH-SY5Y cells decreased from 90.41% to 75.96%. 

However, treatment with 1.0 µmol/L or 10.0 µmol/L GRb1 counteracted the reduction of mitochondrial membrane potential, and maintained it at 81.84% and 87.58%, respectively. This indicated that GRb1 could mitigate OGD-induced reduction in mitochondrial membrane potentials. 

### 2.5. Ginsenoside Rb1 Inhibited Release of AIF and Cyto c from Mitochondria

**Figure 5:**
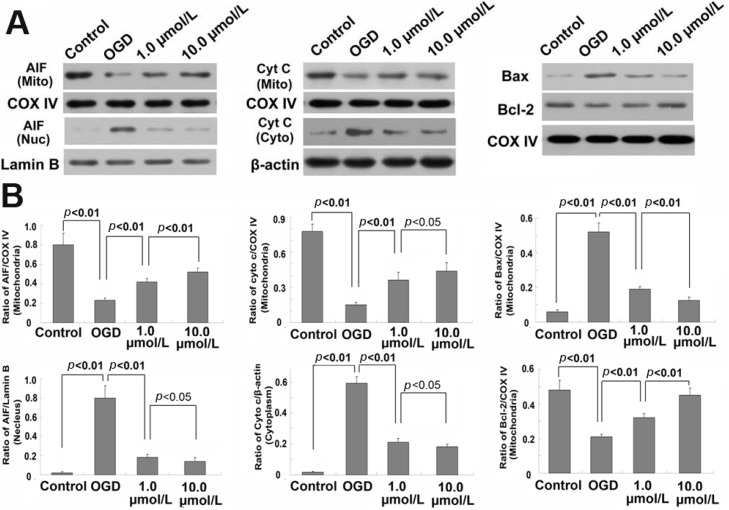
Western blotting analysis of the content of AIF, cyto c, Bax and Bcl-2 within mitochondria, nucleus and cytoplasm. (**A**) representative image of western blotting; (**B**) statistical analysis. * *p* < 0.01 *versus* control group; **^&^** *p* < 0.05 *versus* OGD group; **^#^** *p* < 0.01 *versus* OGD group. This result indicated that the attenuation of GRb1 on the release of AIF and cytochrome c from mitochondria might be via modulating mitochondrial level of Bax and Bcl-2.

**Figure 6:**
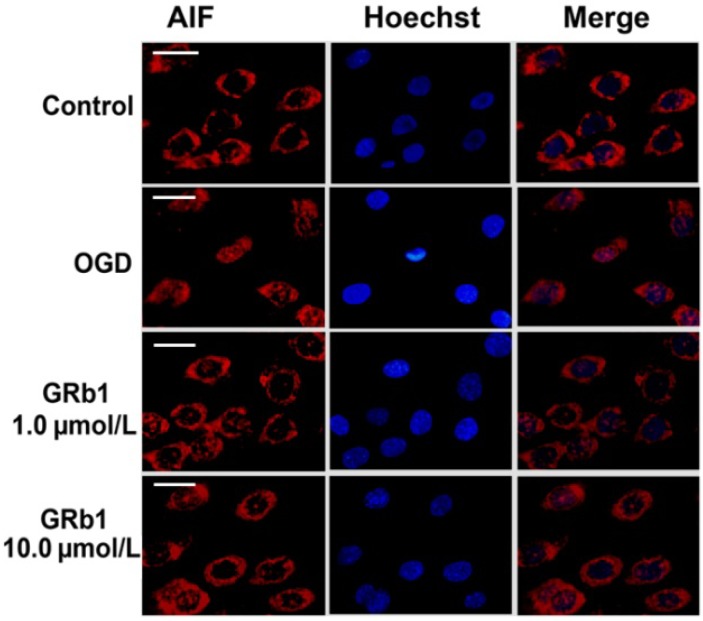
Immunocytochemical analysis of AIF redistribution. Immunocytochemistry showed that part of mitochondrial AIF translocated from mitochondria to nucleus in the OGD treated SH-SY5Y cells, when compared with that in the control group. However, this translocation was suppressed effectively by GRb1 either at the concentration of 1.0 µmol/L or 10.0 µmol/L. Scale bar: 10 µm.

Mitochondrial proteins AIF and cyto c (cytochrome c) play important roles in initiating the intrinsic apoptosis pathway. Thus, we isolated the sub-cellular mitochondrial fraction, nucleus fraction and cytoplasm fraction via differential centrifugation and examined the distribution of AIF and cyto c by western blotting. As shown in Figure 5, compared with that in control group, the levels of AIF and cyto c within mitochondria were reduced significantly in the OGD group, whereas AIF increased within the nucleus and cyto c was elevated in cytoplasm. However, pretreatment with GRb1 either at 1.0 µmol/L or 10.0 µmol/L inhibited the alterations of AIF and cyto c caused by OGD. GRb1 alleviated the reduction of AIF and cyto c within mitochondria, and attenuated the increase of AIF in the nucleus and cyto c in the cytoplasm. Moreover, 10.0 µmol/L GRb1 showed stronger effects than 1.0 µmol/L GRb1 did. Immunocytochemistry demonstrated as well that part of the mitochondrial AIF translocated to the nucleus in OGD treated cells. However, this translocation was suppressed by GRb1 (Figure 6). These results indicated that GRb1 inhibited release of AIF and cyto c from mitochondria. 

### 2.6. Ginsenoside Rb1 Improve Bcl-2 Level, but Attenuated Bax Level Within Mitochondria

Bax and Bcl-2 within mitochondria are both demonstrated to be modulators of the release of AIF and cytochrome c [28]. Thus, we examined the content of Bax and Bcl-2 within mitochondria by western blotting. We found that mitochondrial level of Bax increased, but Bcl-2 decreased in the OGD-treated cells, when compared with those in control group (Figure 6). By contrast, pretreatment with GRb1 inhibited OGD-induced elevation of Bax and the reduction of Bcl-2 within mitochondria. In addition, 10.0 µmol/L GRb1 showed stronger effects than 1.0 µmol/L GRb1 did. This result indicated that GRb1 modulated mitochondrial level Bax and Bcl-2, which might be underlying the mechanism of the inhibitory effects of GRb1 on OGD-induced release of AIF and cyto c. 

## 3. Discussion

The present study showed that GRb1 either at a lower concentration of 1.0 µmol/L or at a higher concentration of 10.0 µmol/L could significantly decrease OGD-induced cell death in SH-SY5Y cells via inhibition of apoptosis. Moreover, we found that the anti-apoptotic effect of GRb1 was associated with its protection on mitochondrial function through suppressing ROS levels, inhibiting mitochondrial membrane potential decline, elevating Bcl-2 but attenuating Bax within mitochondria, and preventing the release of AIF and cyto c from mitochondria. These results indicated that the inhibitory effects of GRb1 on OGD-induced apoptosis in SH-SY5Y cells are via stabilizing mitochondrial function. 

OGD-induced cell death in human SH-SY5Y is often used to imitate the neuronal death caused by cerebral ischemia and reperfusion [29]. Apoptosis, a form of programmed cell death, has been proved to be a common mode of neuronal death caused by various pathological stresses such as traumatic brain injury, subarachnoid hemorrhage and cerebral ischemia and reperfusion [30,31,32]. Thus, prevention or inhibition of apoptosis has become a strategy to protect from neuronal injury. 

Accumulating studies have revealed that GRb1 is an effective agent to inhibit apoptosis. *In vitro* studies showed that GRb1 mitigated apoptosis caused by Herpes Simplex Virus-1 in human U251glioma cells [13], alleviated high glucose/cytokine-induced apoptosis in *Rattus* pancreatic β-cells [33] and suppressed apoptosis due to ultraviolet radiation in HaCaT cells [34]. Consistent with these reports, we demonstrated in this study by using flow cytometry and TUNEL staining that GRb1 could effectively inhibit OGD-induced apoptosis in human SH-SY5Y cells either at lower or higher concentration. Moreover, previous *in vivo* studies using rat models showed as well that GRb1 exerted protective effects against ischemia and reperfusion induced apoptosis in myocardial cells and neurons [11,15]. Thus, both *in vitro* and *in vivo* studies have indicated that GRb1 is an effective protective agent against apoptosis, despite the fact its mechanism is still elusive. 

GRb1 is currently found to exert protection on apoptosis via alleviation of nitric oxide production and down-regulation of the expression of caspase-3 [33], induction of DNA repair [34] and activation ERK1/2 and Akt pathway [15]. However, the mitochondrion is often regarded as a crucial organelle mediating cellular apoptosis. More and more evidence has shown that stabilization of mitochondria could exert protection on brain damage and neuronal apoptosis. Liang *et al*. reported that the protection of ischemic postconditioning on cerebral damage caused by ischemia and reperfusion was via maintaining mitochondrial function [35]. Jiang *et al*. investigated the protective effects of rapamycin on neuronal apoptosis by using a rat model of Parkinson’s disease and found that it was associated with stabilizing mitochondrial function [36]. Agudo-López *et al*. proved that sphingosine-1-phosphate inhibited OGD-induced cell apoptosis through maintaining mitochondrial function [37]. Therefore, *in vivo* and *in vitro* studies have revealed that stabilizing mitochondrial function is a common pathway to reduce neuronal apoptosis. 

Within cells, mitochondria are not only the main source of intracellular reactive oxygen species (ROS), but also the target of ROS attack. ROS attack would result in a decline in mitochondrial membrane potentials, which was often used as an indicator in previous studies to reflect mitochondrial function [38]. Thus, in this study, we examined ROS level and mitochondrial membrane potentials in SH-SY5Y cells. We found pretreatment with GRb1 significantly suppressed the elevation of ROS induced by OGD in SH-SY5Y cells, and mitochondrial membrane potential was maintained at a higher level when compared with that in OGD treated group. Despite we did not investigate the potential mechanism underlying the inhibitory effect of GRb1 on ROS, Lü *et al.* found that Rb1 can significantly and selectively reduce hydroxyl radical (•OH) and hypochlorous acid (HOCl), two of the strongest ROS. Rb1 directly scavenged the •OH and protected plasmid DNA from damage induced by •OH, and inhibited effectively HOCl-induced tyrosine chlorination [39]. Similarly, Li *et al.* proved that GRb1 exhibited potent activities of scavenging hydroxyl radicals [40]. We thus think that part of OGD-induced ROS was cleared by GRb1, leading to lower level of ROS and less attack on the mitochondria. Additionally, Kong *et al.* demonstrated that GRb1 protected cardiomyocytes against CoCl_2_-induced apoptosis in neonatal rats by inhibiting mitochondria permeability transition pore opening [41]. Therefore, both our results and previous studies indicated that the inhibition of GRb1 on apoptosis is associated with protection of mitochondrial function. 

The release of pro-apoptotic proteins AIF and cyto c from mitochondria reflected mitochondrial dysfunction [42]. AIF and cyto c are normally located in the space between the mitochondrial inner membrane and outer membrane. After being released under apoptotic stress, cyto c translocates to the cytoplasm to activate the caspase-dependent apoptotic pathway, and AIF translocates into the nucleus to mediate caspase-independent apoptosis [42]. Culmsee *et al.* found that AIF translocated from mitochondria to nucleus during the course of neuronal injury induced either by cerebral ischemia and reperfusion or by OGD [43]. By contrast, Hu *et al.* reported that blocking AIF mitochondrio-nuclear translocation by ginsenoside Rd benefited neuronal survival after focal cerebral ischemia in rats [44]. Similarly, an *in vitro* study showed as well that suppression of AIF translocation protected epileptic hippocampal neurons from apoptosis [45]. In this study, we demonstrated that GRb1 effectively inhibited OGD-induced translocation of cyto c into the cytoplasm and AIF into the nucleus. This result was consistent with a prior report showing that antioxidants alleviated the release of AIF and cyto c in the ischemia model [46]. Inhibition of the release of AIF and cyto c indicated that pretreatment with GRb1 protected mitochondrial function and inhibited the initiation of caspase-dependent and caspase-independent pathways. Additionally, other researchers have demonstrated as well that GRb1 could prevent apoptosis caused by various inducers. Cai *et al.* reported that ginsenoside Rb1 suppressed apoptosis by indcing expression of specific components of nucleotide excision repair proteins such as XPC and ERCC1 [34]. Hashimoto *et al.* proved that GRb1 attenuated MPP^+^ (1-methyl-4 phenylpyridinium)-induced apoptosis in PC12 cells by stimulating estrogen receptors with consequent activation of ERK1/2, Akt and inhibition of SAPK/JNK, p38 MAPK [47]. Thus, these previous studies all indicated that GRb1 could exert anti-apoptotic effects via multiple pathways. 

Despite the fact that the process of AIF and cyto c release from mitochondria is complicated and still needed to be clarified, it is thought that the release of AIF and cyto c from mitochondria is regulated by pro-apoptotic Bax and anti-apoptotic Bcl-2 [28]. Bax could permeabilize the outer membrane of mitochondria and result in release of AIF and cyto c, but Bcl-2 residing on the outer mitochondrial membrane could block the recruitment of Bax to mitochondria [48]. In this study, we found that the quantity of Bax within mitochondria increased, whereas the Bcl-2 reduced within the SH-SY5Y cells treated with OGD. However, in the SH-SY5Y cells treated with GRb1, the Bax level within mitochondria was markedly lower, but the Bcl-2 level was significantly higher than those in the OGD group. This indicated that GRb1 inhibited the release of AIF and cyto c from mitochondria via modulating mitochondrial level of Bax and Bcl-2. 

## 4. Experimental

### 4.1. Reagents

Ginsenoside Rb1 (purity > 98%) was obtained from the National Institute for the Control of Pharmaceutical and Biological Products (Beijing, China). It was dissolved in physiological saline (0.9% NaCl) at the concentration of 10 mmol/L as stock solutions and was diluted with cell culture media before use. Polycolonal primary antibodies against AIF and β-actin were from Abcam (Cambridge, MA, USA), against Bax, Bcl-2 and Lamin B1 were from Cell signaling (Danvers, MA, USA). Monocolonal primary antibodies against cytochrome c (cyt c) and cytochrome c oxidase IV (COX IV) were from Cell signaling (Danvers). Horseradish peroxidase-conjugated goat anti-rabbit IgG and horse anti-mouse IgG were from Cell signaling (Danvers). ECL Western blotting detection reagents from Amersham Company (Piscataway, NJ, USA). PVDF membranes from Millipore Company (Billerica, MA, USA). Other reagents were from Sigma Company (St. Louis, MO, USA). 

### 4.2. Cell Culture

Human SH-SY5Y cells were obtained from Shanghai Institute of Cell Biology, Chinese Academy of Sciences (Shanghai, China). Cells were cultured in DMEM supplemented with 10% fetal bovine serum, 2 mmol/L glutamine (Gibco, Grand Island, NY, USA), penicillin (100 U/mL) and streptomycin (100 μg/mL), and maintained at 37 °C and 5% CO_2_ in a humid environment. The medium was replaced twice each week.

### 4.3. Cell Viability Assay

SH-SY5Y cells were seeded at a density of 4 × 10^4^ cells per well on collagen-coated 96-well plates for 24 h. The cells were then randomized to the following groups: normal control group (normal SH-SYSY cells were cultured under normoxia for 27 h), OGD group (the cells were treated with 3 h OGD followed by 24 h re-oxygenation), OGD + GRb1 group (the cells were treated with GRb1 at 1.0 µmol/L, 10 µmol/L and 100 µmol/L at 30min prior to OGD and continued to at 24 h after re-oxygenation). Cellular viability was assessed using an MTT assay, and the absorbance value (*A*) at 570 nm was read using an automatic multi-well spectrophotometer (Bio-Rad, Richmond, CA, USA). 

### 4.4. Oxygen-Glucose Deprivation

Oxygen-glucose deprivation followed by re-oxygenation experiments were carried out at 24 h after seeding the cells. SH-SY5Y cells were placed in an anaerobic chamber (HERA cell 150, partial oxygen pressure was maintained below 2 mmHg) and the medium was replaced with a pre-warmed (37 °C) glucose-free balanced salt solution (116 mmol/L NaCl, 5.4 mmol/L KCl, 0.8 mmol/L MgSO_4_, 1.0 mmol/L NaH_2_PO_4_, 1.8 mmol/L CaCl_2_, 26.2 mmol/L NaHCO_3_, 0.025 mmol/L phenol red, and 20 mmol/L sucrose) that had been bubbled with an anaerobic gas mix (95% N_2_, 5% CO_2_) for 30 min to remove residual oxygen. SH-SY5Y cells were incubated in this solution at 37 °C for 3 h to produce lethal oxygen-glucose deprivation. OGD was terminated by removing cells from the chamber, replacing the exposure solution with normal DMEM culture, and returning to the incubator under normoxic conditions. 

### 4.5. Assessment of Apoptosis by TUNEL Staining

Apoptosis was further demonstrated by TUNEL staining. In brief, SH-SY5Y cells were washed with PBS and fixed in 4.0% paraformaldehyde for 20 min at room temperature. After washed three times in PBS buffer, the fixed cells were subjected to a TUNEL assay by using an *in situ* cell death detection kit (Roche, Indianapolis, IN, USA) according to the manufacturer’s specifications. Finally, nucleus counterstaining was performed by incubating cells with propidium iodide at room temperature for 10 min. The cells were mounted on slides after being washed three times with PBS, and visualized under a fluorescent light microscope. Green fluorescence was correlated with DNA fragmentation. The cells with green fluorescence and the cells with red fluorescence were counted to quantify the apoptotic process. Data are expressed as the ratio of TUNEL-positive cells to total cells. Experiments were done in duplicate for three times, and the percentage of TUNEL-positive cells was determined. 

### 4.6. Detection of Apoptosis by Flow Cytometry

Cellular apoptosis was evaluated by using annexin V-FITC apoptosis detection kit (Invitrogen, Grand Island, NY, USA) and flow cytometry. Briefly, SH-SY5Y cells were collected at 24 h after re-oxygenation, washed twice with PBS and subjected to Annexin V-FITC and propidium iodide (PI) double staining as described by the manufacture’s instruction. Finally, the stained cells were analyzed by flow cytometry (FACScan, Becton Dickinson, San Jose, CA, USA). The rate of cell apoptosis was analyzed using CELLquest software (Becton Dickinson). Data acquisition was conducted by collecting 20,000 cells per tube and the numbers of viable and dead cells were determined for each experimental condition.

### 4.7. Measurement of Intracellular ROS Levels

The average level of intracellular ROS was evaluated in cells loaded with the redox-sensitive dye DCFH-DA (Molecular Probes, Eugene, OR, USA). All the experimental cells were washed twice with PBS, stained in the dark for 30 min with 20 μmol/L DCFH-DA and harvested. Cells were dissolved with 1% Triton X-100, and fluorescence was observed under fluorescent light microscope, and measured at an excitation wavelength of 485 nm and an emission wavelength 530 nm using a fluorescence spectrometer (HTS 7000, Perkin Elmer, Boston, MA, USA). The ROS levels were expressed as arbitrary unit/mg protein, then as the percentage of control.

### 4.8. Mitochondrial Membrane Potential

Mitochondrial membrane potential (ΔΨm) was determined by the retention of the dye rhodamine 123. The collected cells were washed twice with PBS. After incubation with 10 μg/mL rhodamine 123 at 37 °C for 30 min, the cells were washed again and fluorescence intensities of Rhodamine 123 in cells were analyzed with flow cytometry (FACScan, Becton Dickinson). 

### 4.9. Immunofluorescence Staining

The collected SH-SY5Y cells were washed with PBS and fixed in 4.0% paraformaldehyde for 30 min at 4 °C. After washing the cells with PBS three times for 5 min each time, cells were incubated with 1% Triton X-100 for 10 min. The cells were blocked at nonspecific antibody binding sites by incubating with 10% goat serum in PBS containing 0.3% Triton X-100 and 0.5% BSA (bovine serum albumin) for 10 min at room temperature. Cells were then incubated with a mouse monoclonal antibody against AIF (1:200 in PBS) followed by incubation in TRIC-conjugated goat anti-mouse IgG (1:150 in PBS) for 30 min at room temperature. After washing with PBS, cells were incubated with Heochst33342 for 0.5 h. After washing three times, the cells were mounted on slides cells and were visualized under fluorescence microscopy. 

### 4.10. Differential Centrifugation and Cellular Fraction

The collected cells were washed with PBS, and centrifuged 10 min at 1,000 ×*g*, the cell pellets were suspended in ice-cold lysis buffer containing 15 mmol/L Tris, pH7.6, 250 mmol/L sucrose, 1 mmol/L MgCl_2_, 2.5 mmol/L EDTA, 1 mmol/L EGTA (ethylene glycol-bis(β-aminoethylether) tetraacetic acid), 1 mmol/L dithiothreitol, 1.25 mg/mL pepstatin A, 10 mg/mL leupeptin, 2.5 mg/mL aprotinin, 1.0 mmol/L phenylmethylsulfonyl fluoride (PMSF), 0.1 mmol/L Na_3_VO_4_, 50 mmol/L NaF, and 2 mmol/L Na_4_P_2_O_7_ and homogenated with a glass Pyrex microhomogenizer (20 strokes). The homogenates were centrifuged at 800 ×*g* at 4 °C for 10 min to obtain the pellet containing nucleus fraction. The supernatants were then centrifuged at 15,000 ×*g* at 4 °C for 10 min to obtain the pellet containing mitochondria and the supernatant containing cytosolic fraction. All the pellets were re-suspended in lysis buffer. The protein content of each cellular fraction was determined using Bio-Rad protein assay kit.

### 4.11. Gel Electrophoresis and Western Blotting

Equal protein amounts were electrophoresed on 10% sodium dodecyl sulfate-polyacrylamide gels and then transferred to PVDF membranes. The membranes were blocked with 3% bovine serum albumin in TBS for 30 min and then incubated respectively overnight at 4 °C with the following primary antibodies against AIF (1:1,000), Bax (1:1,000), Bcl-2 (1:1,000), β-actin (1:3,000), Lamin B1 (1:1,000), cytochrome c (cyt c) (1:1,000) and cytochrome c oxidase IV (COX IV) (1:1,000). After being incubated with horseradish peroxidase-conjugated goat anti-rabbit IgG (1:2,000) or horse anti-mouse IgG (1:2,000), blots were washed and immunoreactive proteins were visualized on a Kodak X-omat LS film (Eastman Kodak Company, New Haven, CT, USA) with an enhanced chemiluminescence. Densitometry was performed with Kodak ID image analyses software (Eastman Kodak Company).

### 4.12. Statistical Analysis

All data represent at least four independent experiments and are expressed as mean ± SD. Statistical comparisons were made using one-way ANOVA. *p*-values of less than 0.05 were considered to represent statistical significance. 

## 5. Conclusions

In this study, we demonstrated that the inhibition of GRb1 on OGD-induced apoptosis in SH-SY5Y cells is via modulating mitochondrial function, including maintaining mitochondrial membrane potential, suppressing the translocation of AIF to nucleus and cytochrome c to cytoplasm, elevating mitochondrial level of anti-apoptotic Bcl-2 and reducing pro-apoptotic Bax within the mitochondria. This might be helpful to understand the mechanism underlying the anti-apoptotic effects of GRb1.
